# Incidence and Survival of Pediatric Soft Tissue Sarcomas in Moscow Region, Russian Federation, 2000–2009

**DOI:** 10.1155/2012/350806

**Published:** 2012-04-04

**Authors:** D. Y. Kachanov, K. V. Dobrenkov, R. T. Abdullaev, T. V. Shamanskaya, S. R. Varfolomeeva

**Affiliations:** ^1^Department of Oncology and Hematology, Russian State Medical University, 1 Ostrovityanova Street, Moscow 117997, Russia; ^2^Department of Clinical Oncology, Federal Research Center of Pediatric Hematology, Oncology and Immunology, 1 Samory Mashela Street, Moscow 117198, Russia

## Abstract

The aim of the study was to assess the incidence and survival rates of soft tissue sarcomas (STSs) in children 0–14 years of age in Moscow Region, Russian Federation. The database of childhood population-based cancer registry of Moscow Region was used as a data source. Tumors were stratified according to International Classification of Childhood Cancer, 3d ed. Sixty-eight cases of STS were registered from 2000 to 2009. Crude incidence rate was 0,78, and age-standardized incidence rate using World Standard Population was 0,81 per 100.000 children/year. The highest age-specific incidence was observed in infants: 1,76 per 100.000 children/year. Rhabdomyosarcoma (RMS) was the most common histological type comprising 54,4% of all STS. 5-year observed survival (OS) of all patients with STS was 64,1 (95% CI 55,0–73,2). There was no statistically significant difference in OS between RMS—59,2 (95% CI 47,0–71,4) and nonrhabdomyosarcoma STS—69,3 (95% CI 55,8–82,8) (*P* = 0.63). Incidence and survival rates of STS observed in the study were comparable to the other Eastern European countries.

## 1. Introduction

Soft tissue sarcomas (STSs) represent a heterogeneous group of neoplasms originating from primitive mesenchymal cells [1]. Population-based data suggest that these tumors comprise 4–8% of all cancer cases in patients under the age of 15 years [2–4]. STSs represent the second most common extracranial solid tumor in children in Europe and the USA following neuroblastoma [3, 4]. STSs are divided into two distinct groups: rhabdomyosarcoma (RMS) and nonrhabdomyosarcoma STS (NRSTS) [1]. RMS is the single most frequent childhood STS, accounting for more than 50% of cases. NRSTS represents biologically and clinically heterogeneous assortment tumors. Distribution of subgroups of STS is age dependent. RMS is more common in children less than 10 years of age whereas NRSTS predominates in older age groups [1].

Large international epidemiological studies have shown that the incidence of STS varies in different geographic regions and ethnic groups [2]. STS epidemiology in children in Russian Federation (RF) has not been adequately studied due to several reasons. The nationwide system of cancer registration established in RF in 1953 stratifies malignancies by topography of primary tumor. International classifications specifically designed for children (e.g., International Classification of Childhood Cancer, 3d eds.—ICCC-3) and based on the histogenesis of tumors are not currently used for data analysis and reporting. At the same time STS in childhood can occur in a wide range of primary sites and are of many histological types. Moreover, there is a lack of information on the survival rates of children with cancer in RF. In order to overcome these limitations a childhood population-based cancer registry was established in Moscow Region (MR) in 2000.

The aim of this study is to describe incidence and survival rates of STS in children 0–14 years old in MR for the period from 2000 to 2009. The data were analyzed for the whole group of STS and separately for RMS and NRSTS subgroups.

## 2. Materials and Methods

Data on patients were retrieved from the database of childhood population-based cancer registry of MR that has prospectively registered all cancer cases in children 0–14 years old since 2000. MR is located in the central part of RF and occupies 46.000 km^2^ with total population of 6,6 million (2008). The region is divided in 72 municipal districts (okrug) which are clustered in 12 medical districts. Moscow city is not an administrative part of the region, and its territory is not covered by the cancer registry of MR.

All patients 0–14 years old with STS diagnosed in 2000–2009 were included in analysis. Case finding of patients diagnosed before 2000 allowed to exclude prevalent cases and include in the final analysis patients with STS diagnosed in 2000. The lag time from diagnosis to recording in the registry was estimated to be less than 1 year.

Multiple sources of information on new cancer cases were used and included but not limited to discharge letters from cancer hospitals, data from pathology laboratories, and from general cancer registry of Moscow Region. By the national law oncologists are obliged to fill a notification form on every newly diagnosed case of cancer. The cancer registry has an access to the database of Ministry of Healthcare of Moscow Region that contains information on patients referred to federal cancer centers. The cancer registry closely collaborates with primary care pediatricians. Notification forms for newly diagnosed patients and for patients who finished anticancer therapy were specially designed for data retrieval. These forms represent a legal document of the mandatory annual report from pediatricians of municipal districts. Follow-up information was collected by active search in hospital databases and by passive retrieval of notification form filled by primary care pediatricians. Identifiable death certificates were not available to the registry. Depersonalized cancer mortality database of MR was used, and special requests are sent to primary care pediatricians to trace back other records for these patients. The completeness of registration exceeded 90%.

For each registered case, information included civil status profile (name, surname, gender, date of birth, and address at the time of cancer diagnosis) and disease profile (incidence date, primary site and histology, diagnostic basis, stage, and type of therapy). Diagnoses were confirmed by reference pathology laboratory in all cases. None of the cases were registered from death certificate only. Multiple primary tumors in the same patient were separately registered. The localization of the primary tumor and histological type was defined in accordance to International Classification of Diseases for Oncology (ICD-O), 3rd edition [5]. Subsequently, tumors were grouped according to International Classification of Childhood Cancer, 3rd ed. (ICCC-3) [6]. ICCC-3 refers STS to IX diagnostic group divided into 5 subgroups: IXa: RMS, IXb: fibrosarcomas, peripheral nerve sheath tumors, and other fibrous neoplasms (further in text-subgroup of “fibrosarcoma”), IXc: Kaposi sarcoma, IXd: other specified STS, and IXe: unspecified STS. For further analysis, STSs were divided into RMS (IXa subgroup) and NRSTS (IXb, IXc, IXd, and IXe subgroups) [6].

For classification of the primary tumor localization, ICD-O-3 topography code was grouped in 8 sites [7] (Table 1).

Most of the registered patients (45/68, 66,2%) were treated according to consecutive protocols of the Cooperative Soft Tissue Sarcoma Study Group (Cooperative Weichteilsarkom-Studie, CWS) of the German Society of Pediatric Oncology and Haematology (GPOH) [8]. The rest of the patients received anticancer treatment according to institutional protocols that included multiagent chemotherapy, radiation therapy, and surgery.

Annual population estimates in MR were based on the data of RF population census in 2002 and were received from Moscow Regional Committee of Federal State Statistics Service. The average annual population in the given period was 876.695 (95% CI: 843.717 to 909.673) for children aged 0–14 years. A decrease in childhood population from 976.795 in 2000 to 868.932 in 2009 was observed during the study period. Crude annual incidence rate was calculated per 100.000 children/year. Age-standardized incidence rate (ASR) was calculated by direct method per 100.000 children/year using the World Standard Population [9]. Crude incidence rate and ASR were calculated for children 0–14 years old. Age-specific incidence rate was calculated for four different age groups: <1, 1–4, 5–9, and 10–14 years. Kaplan-Meier method was used for survival analyses [10]. Observed survival was calculated for the entire group of patients, for the age groups 0–4, 5–9, 10–14 years, and separately for patients with RMS and NRSTS. Differences in survival rates between various patient groups were evaluated by comparison of entire survival curves and tested with the log-rank tests. Fisher's exact test was used to compare categorical variables due to small number of cases. Statistica 8.0 (Statsoft, USA) was used for statistical analysis of the data.

## 3. Results

Sixty-eight cases of STS in children 0–14 years old were registered in 2000–2009, comprising 5,8% of all malignant tumors in this age group. STS was the third most frequent extracranial solid tumor in children after neuroblastoma and renal tumors. There was a slight male prevalence with the male-to-female ratio which was 1,52 : 1. Most cases were observed in 10–14 age group (22 patients, 32,4%). 20 (29,4%) and 16 (23,5%) cases were registered in 1–4 and 5–9 age groups, respectively. The smallest number of patients was registered in infants—10 (14,7%) cases. The median age at diagnosis was 5 years.

Distribution of STS by diagnostic subgroup according to ICCC-3 showed the prevalence of subgroup “RMS” (Table 2), followed by subgroups of “other specified STS” and “fibrosarcoma”. Kaposi sarcoma cases were not identified.

RMS was the most common histological type of STS, representing 54,4% of cases. Among NRSTS there were 8 (11,8%) cases of sinovial sarcoma, 7 (10,3%) malignant peripheral nerve sheath tumor, and 5 (7,4%) cases of Ewing sarcoma family. Analysis of distribution of STS cases according to age demonstrated a negative correlation between number of RMS and patient's age: RMS comprised 70% of STS cases in infants and 80% in children 1–4 years of age, but only 23% in 10–14-year age group. 

Location of primary tumor differed between RMS and NRSTS. Majority of RMS (43.3%) was located in the head and neck region (including orbit) whereas NRSTS mainly affected limbs (45.2%) (Table 3). 

The crude incidence rate of STS in children 0–14 years old was 0,78 ASR was 0,81 per 100.000 children/year (Table 2). The highest age-specific incidence rate was observed in infants—1,76 per 100.000 children/year. Incidence rate of STS subgroups varied in different age groups. Age-specific incidence rate of RMS (IXa) was the highest in infants, decreasing in older age groups. Incidence rate of “other specified STS” (IXd) showed bimodal distribution with the highest incidence observed in infants and children 10–14 years old. 

Cancer predisposition syndromes presented by neurofibromatosis type 1 were observed in 3 (4,4%) cases. In 1 case RMS developed as a second malignant neoplasm in the radiation field 3,4 years after the diagnosis of hereditary retinoblastoma. 

**Figure 1:**
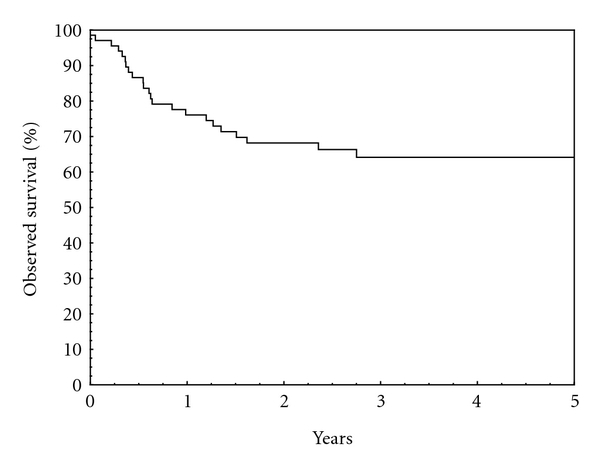
Observed survival, all patients. OS = 64,1 (95% CI 55,0–73,2) at 5 years.

The OS of all patients with STS at 5 years was 64,1 (95% CI 55,0–73,2) (Figure 1). 

**Figure 2:**
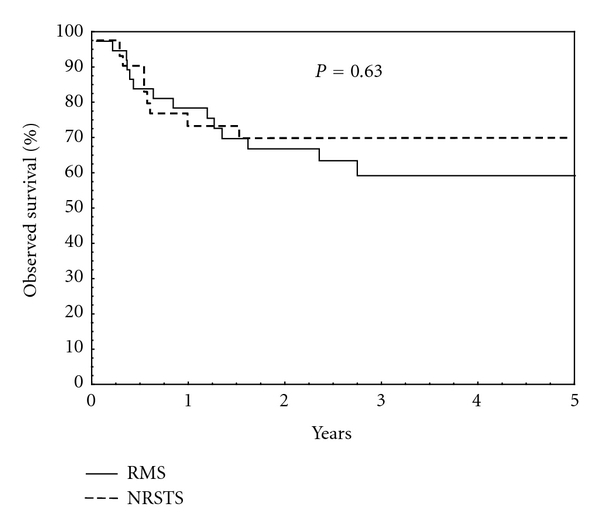
Observed survival, RMS versus NRSTS. OS for RMS = 59,2 (95% CI 47,0–71,4) at 5 years. OS for NRSTS = 69,3 (95% CI 55,8–82,8).

There was no statistically significant difference in 5-year OS between RMS—59,2 (95% CI 47,0–71,4) and NRSTS—69,3 (95% CI 55,8–82,8) (*P* = 0,63) (Figure 2). 

The better 5-year OS rate was observed in patient 5–9 years of age 80,0% (95% CI 62,5–97,5). The 5-year OS for patients 0–4 and 10–14 years of age was 58,3% (95% CI 44,8–71,8), and 62,3% (95% CI 46,3–78,3), respectively. Five-year OS in age groups <1, 1–9 and >10 years was 50,0% (95% CI 28,1–71,9), 70,0% (95% CI 57,5–82,5) and 62,2% (95% CI 46,3–78,1), respectively. Difference in survival between age groups was not statistically significant. 

## 4. Discussion 

The present study describes the incidence and survival of STS in children in MR, the second most populated region of RF. Epidemiology studies of childhood cancer are hampered by the lack of population-based cancer registries in Russia. This study is the first published report presenting detailed analysis of epidemiological characteristics of STS in children in RF. Childhood population-based cancer registry data were used for analysis that precludes selection bias, typical for hospital-based studies. 

Children <15 years of age were included. It should be noted that in the literature different age intervals for the definition of “pediatric” population are used. Ferrari et al. [11] use this term for cases aged <20 years. On the contrary, Automated Childhood Cancer Information System (ACCIS) in Europe separately analyzes cases in children 0–14 years old and adolescents 15–19 years old [12]. 

The incidence of STS in children varies considerably depending on geographic region and ethnic group [2]. In general, higher incidence rates were observed in Europe and North America, lower ones in Asian countries. Data from the International Agency for Research on Cancer (IARC) showed that incidence rate of STS in Caucasians children varied from 0,5 to 0,9 per 100.000 per year [2]. According to ACCIS average incidence rate of STS in children <15 years in Europe is 0.91 per 100.000 per year, and it varies between 5 geographic regions [12]. The highest incidence rate of STS is observed in Northern countries (1,1 per 100.000 children per year), the lowest in Eastern Europe (0.84 per 100.000 children per year). The cause of these geographical differences is not clear; however, the impact of variations in diagnostic, classification, and registration criteria could not be ruled out [12]. The incidence rate of STS obtained in our study (0,81 per 100.000 children per year) was comparable to the data of the Eastern European countries included in ACCIS project. It reflects the completeness of childhood STS registration by childhood population-based cancer registry in MR. 

Certain congenital anomalies and genetic conditions (e.g., Li-Fraumeni syndrome, neurofibromatosis type I) are known as the strongest risk factors, although they explain only a small proportion of cases [3, 13]. Thus, Narod et al. reported cancer predisposition syndromes in 20 (2,0%) out of 1003 children with STS in Great Britain [14]. Radiation therapy is the best-known environmental risk factor for STS [15]. HIV infection is responsible for cases of Kaposi's sarcoma in Africa [2]. Kaposi's sarcoma makes up about two-thirds of STS and 20% of all childhood cancers in some African countries [16] but only 1% of STS in the US [3]. 

In our study genetic predisposition syndromes were revealed in 4,4% cases and were presented by neurofibromatosis type 1. In 1 patient with hereditary retinoblastoma RMS developed as a second malignant neoplasm in the radiation field. No cases of Kaposi's sarcoma in our study were detected, which is consistent with the data of the European and North American cancer registries, indicating extreme rarity of the disease [3, 12]. 

The incidence rate of STS in children varies by age. The highest incidence seen in infants (1,3–2,1 per 100.000) gradually decreases to 0,6–0,8 per 100.000 per year in 5–9 year age group, with further increase to 0,72–1,1 per 100.000 per year in children older than 10 years [3, 12]. The results of our study also corroborate with the previous findings: the highest incidence observed in infants (1,76 per 100.000 per year), the lowest in 5–9-year age group (0,61 per 100.000 per year). The biological basis of age-dependent differences in incidence rate is not clear. It is speculated that higher STS incidence in young children could be a result of genetic predisposition. Diller et al. in a study of 33 cases of sporadic RMS showed that 3 of 13 children younger than 3 years of age at diagnosis (compared with none of the 20 children older than 3 years of age) had germline mutations in their p53 gene [17]. 

The distribution of specific subgroups of STS according to ICCC-3 showed prevalence of RMS (IXa) and the subgroup of “other specified STS” (IXd). The most extensive studies on STS epidemiology in children [2, 3, 12] were based on International Classification of Childhood Cancer, 2d ed. (ICCC-2) [18]. Several histological types (e.g., pleomorphic malignant fibrous histiocytoma) assigned to a subgroup of “fibrosarcoma” (IXb) in ICCC-2 were included into subgroup of “other specified STS” (IXd) [6]. It complicates a comparative analysis of different STS subgroups because in our study tumors were stratified according to ICCC-3. Nevertheless, recent report of German Childhood Cancer Registry based on ICCC-3 [7] confirmed our findings. RMS and “other specified STS” were the two most common groups of STS in children comprising 58,3% and 27,3%, respectively [7]. RMS represents the single most common histological type of STS in our childhood population. This observation is consistent with the population-based data from other countries [3, 7]. Age-related changes in ratio of RMS and NRSTS identified in children in MR have been previously described by others [3, 7, 12]. Data from German Childhood Cancer Registry showed decreased percentage of RMS in older age groups: 71, 64 and 34% in children 0–4, 5–9, and 10–14 years of age, respectively [7]. Correlation of histological tumor type with the topography of primary tumor is well known [1]. As expected the most frequent localization of RMS was the head and neck region, whereas NRSTS predominantly affected limbs. 

Analysis of survival of children with cancer at the population level is an extremely important index reflecting the quality of specialized medical care. Data from ACCIS project showed significant differences in survival of children with STS in Europe [12]. Thus, for the pooled European data 5-year survival of patients with STS diagnosed in 1988–1997 years was 65%, ranging from 74% in Northern and Southern Europe to 50% in Eastern Europe [12]. The average survival rate of children with RMS in Europe was 63%, with the lowest survival observed in Eastern Europe—40%. In the recently published report of Eurocare project 5-year survival of children with RMS in Eastern Europe was 64,9% [19]. Data from the USA Surveillance Epidemiology and End Results program (SEER) showed 5-year survival rate of patients registered in 2001–2007, 72,9% for the entire group of STS, and 68.1% for RMS [20]. 5-year survival of children with STS, obtained in this study, was 64,1% for the whole group of STS and 59,2% for RMS and was lower in Western countries but compatible to the data from East Europe. It should be noted that STS represents significant challenges for treatment and requires a multidisciplinary approach to therapy. Protocol-based therapy of cancer in children, including STS, was introduced in MR only in 2000, that definitely has affected treatment results obtained in the present study. Age is an important prognostic factor in STS, especially in RMS [1, 11, 12, 21]. The worst results of therapy have been reported in infants and children over 10 years of age at the time of diagnosis. According to ACCIS data the better survival was observed in children aged 5–9-year (70%), whereas in other age groups survival ranged from 57% in infants up to 64% in children aged 1–4 years [12]. In our study there was a trend towards better survival of children in 5–9 years age group (5-year OS 80%), but difference was not statistically significant due to small number of patients. 

In conclusion, the present study provides the first data on incidence and survival of STS in children in RF. The epidemiological pattern of the disease is compatible with the data from Europe and North America. Small number of patients is one of the main limitations of this study. On the contrary, strength of this analysis includes population-based data and accuracy of the data confirmed by microscopic verification in all cases, lack of death certificate cases, and low number of patients assigned to the subgroup “unspecified STS”. The obtained data can form a basis for development of population cancer control programs for children with cancer in other regions of RF.

## Figures and Tables

**Table 1:** Definition of the topographic categories of STS.

Site of primary tumor	ICD-O-3 topography code [5]
Head and neck	C00.0–C14.8, C30.0–C32.9, C41.0–C41.1, C44.0, C44.2–C44.4, C47.0, C49.0, C76.0
Orbit	C69.0–C69.9, C44.1
Pelvis	C41.4, C47.5, C49.5, C76.3
Genitourinary system	C51.0–C57.9, C60.0–C60.9, C61.9, C62.0–C63.9, C64.9–C66.9, C67.0–C67.9, C68.0–C68.9
Thorax	C49.3, C76.1
Limbs	C40.0–C40.9, C44.6, C44.7, C47.1, C47.2, C49.1, C49.2, C76.4, C76.5
Not otherwise specified	C76.2, C76.7, C76.8, C80.9 and C49.9
Others	All codes not listed in another category

**Table 2:** Absolute number of cases, crude, ASR, and age-specific incidence rate of soft tissue sarcomas by ICCC-3 in children (0–14 years) diagnosed in Moscow Region in 2000–2009.

	*N*	%	Incidence rate (per 100.000 children per year)						M/F ratio
			Age specific				Crude^1^	ASR	
			<1	1–4	5–9	10–14			
IX soft tissue sarcomas	68	100	1,76	0,95	0,61	0,63	0,78	0,81	1,52
IXa rhabdomyosarcomas	37	54,40	1,23	0,76	0,35	0,14	0,42	0,48	2,08
IXb fibrosarcoma	8	11,80	0,00	0,00	0,12	0,14	0,09	0,08	1,67
IXc kaposi sarcoma	0	0,00	0,00	0,00	0,00	0,00	0,00	0,00	0,00
IXd other specified STS	22	32,40	0,35	0,19	0,15	0,34	0,25	0,24	1,00
IXe unspecified STS	1	1,40	0,18	0,00	0,00	0,00	0,01	0,01	0,00

^1^The sum does not correspond to 0,78 due to rounding in calculations.

**Table 3:** Frequency and percentage of STS by site and histology.

Site of tumor	All STS		RMS		NRSTS		RMS versus NRSTS
	*N*	%	*N*	%	*N*	%	(Fisher's exact test)
Head and neck	17	25,0	11	29,8	6	19,3	n.s.^2^
Orbit	5	7,3	5	13,5	0	0	n.s.
Pelvis	8	11,8	6	16,2	2	6,5	n.s.
Genitourinary system	6	8,8	6	16,2	0	0	*P* = 0,0279
Limbs	17	25,0	3	8,1	14	45,2	*P* = 0,0005
Thorax	4	5,9	1	2,7	3	9,7	n.s.
Others	10	14,7	4	10,8	6	19,3	n.s.
NOS^1^	1	1,5	1	2,7	0	0	n.s.

Total	68	100,0	37	100,0	31	100,0	

^1^NOS: non otherwise specified; ^2^n.s.: not significant.
